# Is Systemic Immune-Inflammation Index a Real Non-Invasive Biomarker to Predict Oncological Outcomes in Patients Eligible for Radical Cystectomy?

**DOI:** 10.3390/medicina59122063

**Published:** 2023-11-22

**Authors:** Pierluigi Russo, Filippo Marino, Francesco Rossi, Francesco Pio Bizzarri, Mauro Ragonese, Francesco Dibitetto, Giovanni Battista Filomena, Denise Pires Marafon, Chiara Ciccarese, Roberto Iacovelli, Savio Domenico Pandolfo, Achille Aveta, Simone Cilio, Luigi Napolitano, Nazario Foschi

**Affiliations:** 1 Department of Urology, Fondazione Policlinico Universitario Agostino Gemelli, Largo Francesco Vito 1, 00168 Rome, Italy or pierluigi.russo@radboudumc.nl (P.R.); francescorossi1010@gmail.com (F.R.); francescopiobizzarri@gmail.com (F.P.B.); mauroragonese@yahoo.it (M.R.); francescodibitetto@gmail.com (F.D.); giovanni.filomena17@gmail.com (G.B.F.); nazario.foschi@policlinicogemelli.it (N.F.); 2 Department of Urology, Radboud University Medical Center, 6525 GA Nijmegen, The Netherlands; 3 Section of Hygiene, Department of Life Sciences and Public Health, Università Cattolica del Sacro Cuore, 20123 Milano, Italy; 4 Department of Medical Oncology, Fondazione Policlinico Universitario Agostino Gemelli, Largo Francesco Vito 1, 00168 Rome, Italy; ciccarese.c@gmail.com (C.C.); roberto.iacovelli@policlinicogemelli.it (R.I.); 5 Division of Urology, AORN “San Giuseppe Moscati”, 83100 Avellino, Italy; pandolfosavio@gmail.com (S.D.P.); achille-aveta@hotmail.it (A.A.); simocilio.av@gmail.com (S.C.); nluigi89@libero.it (L.N.)

**Keywords:** SII, biomarkers, transitional cell carcinoma, inflammation, prognosis

## Abstract

*Background and Objectives*: To assess the potential prognostic role of the systemic immune-inflammation index (SII) in predicting oncological outcomes in a cohort of patients treated with radical cystectomy (RC). *Materials and Methods*: From 2016 to 2022, a retrospective monocentric study enrolled 193 patients who were divided into two groups based on their SII levels using the optimal cutoff determined by the Youden index. The SII was obtained from a preoperative blood test approximately one month before RC. Univariable and multivariable logistic regression analyses were conducted to investigate the capacity of SII to predict lymph node invasion (N), advanced pT stage (pT3/pT4), and locally advanced condition at the time of RC. Multivariable Cox regression models adjusted for preoperative and postoperative features were used to analyze the prognostic effect of SII on recurrence-free survival (RFS), cancer-specific survival (CSS), and overall survival (OS). *Results*: The optimal cutoff value of the SII was 640.27. An elevated SII was seen in 113 (58.5%) patients. Using the multivariable preoperative logistic regression models, an elevated SII was correlated with nodal invasion (N; *p* = 0.03), advanced pT stage (*p* = 0.04), and locally advanced disease (*p* = 0.005), with enhancement of AUCs for predicting locally advanced disease (*p* = 0.04). In multivariable Cox regression models that considered preoperative clinicopathologic factors, an elevated SII was linked to poorer RFS (*p* = 0.005) and OS (*p* = 0.01). Moreover, on multivariable Cox regression postoperative models, a high SII was linked to RFS (*p* = 0.004) and to OS (*p* = 0.01). *Conclusions*: In this monocentric retrospective study, higher preoperative SII values predicted worse oncological outcomes in patients with bladder cancer (BCa) who underwent RC.

## 1. Introduction

Worldwide, bladder cancer (BCa) is one of the most common tumors; it is in tenth place in both sexes and in seventh place in males [1]. Most patients (80%) with BCa present with a superficial disease or non-muscle invasive bladder cancer (NMIBC), and the remaining part (20%) present with muscle-invasive bladder cancer (MIBC). Patients with NMIBC can be divided into low and intermediate groups, with recurrence-free survival (RFS) rates of 43% and 33%, respectively. About 21% of high-risk NMIBC patients progress to MIBC [2]. The standard treatment for patients with the highest risk for bacillus Calmette–Guerin-unresponsive NMIBC, urothelial MIBC, and MIBC with variant histologies is radical cystectomy (RC) accompanied by extended pelvic lymph node dissection [3,4]. Nevertheless, RC yields a 5-year survival rate of approximately 50% in patients [5,6,7]. These outcomes are also influenced by the pathological stage and clinical prognosis. While notable advancements in treatment modalities, such as targeted therapy, antibody-drug conjugates, and checkpoint inhibition immunotherapy, have emerged recently, BCa still exhibits unfavorable prognoses and clinical outcomes due to local recurrence and distant metastasis. As advancements in medical technology and our understanding of bladder cancer continue to progress, the imperative to identify high-risk patients post radical cystectomy has become increasingly evident. However, the current context of risk assessment in BCa presents several obstacles, and the consequences of not surmounting these challenges are extensive, which can impact not just the outcomes of single patients but also healthcare resource allocation and the overall quality of bladder cancer management. Recent evidence suggests that the modulation of the immune system in cancer patients during tumor development and progression has interesting connections [8,9].

Different prognostic indicators, including C-reactive protein (CRP), systemic inflammation score (SIS), platelet-to-lymphocyte ratio (PLR), and lymphocyte-to-monocyte ratio (LMR), have been reported to be able to predict the prognosis of patients with BCa [10,11,12].

Also, NLR (neutrophil-to-lymphocyte ratio) is a biomarker that is used for cancer prognosis. An intensified neutrophil response and/or the suppression of lymphocytes, which can both result in a high NLR, could potentially facilitate carcinogenesis and impede the anti-tumor immune response. A meta-analysis of 17 articles showed that an elevated NLR is associated with worse overall survival (OS), cancer-specific survival (CSS), and recurrence- free survival (RFS) in renal cell carcinoma and also in bladder cancer [13].

The pan-immune inflammation value (PIV), an innovative biomarker that incorporates counts of neutrophils, platelets, monocytes, and lymphocytes, has demonstrated robust predictive capability for survival outcomes. Notably, it outperforms other widely recognized immune-inflammatory biomarkers in patients diagnosed with colorectal cancer and breast cancer, and even in Bca, its high value is associated with poorer OS and disease-free survival (DFS) [14].

The systemic immune-inflammation index (SII), which is computed by neutrophil count × platelets count/lymphocyte count, is an immune and inflammatory index that reflects the patient’s systemic inflammatory status because it combines three immune cells in a single measurement and was linked to poor outcomes in several types of cancer and urologic cancers [15,16,17,18]. Notably, the existing body of literature on the association between the SII index and oncologic outcomes in BCa patients has produced a spectrum of results, revealing a complex and often inconclusive landscape. Recognizing these disparities in the findings, we sought to address this variability by conducting a focused cohort analysis and aimed to offer crucial information about the potential correlation between the SII index and poor oncologic outcomes in a standardized and well-defined patient cohort. Our monocentric and retrospective study aimed to test the role of the SII as a potential preoperative predictor of aggressive disease and poor outcomes.

## 2. Materials and Methods

### 2.1. Patients and Study Methodology

The medical dossiers of 314 patients with non-metastatic UBC and no other histological variants who underwent RC at the author’s hospital (one institution) between January 2016 and November 2022 were reviewed retrospectively. The data collection process was performed in observance of the guidelines outlined in the declaration of Helsinki following approval from the Institution’s Ethical Committee.

Patients undergoing neoadjuvant chemotherapy (115) and those with autoimmune disease (2), history of radiation therapy of the pelvis (3), and history of combination surgery (1) were excluded. A total of 193 patients were enrolled in this retrospective study. All patients underwent open and robotic RC and nodal dissection. The extent of lymph node dissection and type of urinary diversion were dependent on the patient’s clinical features and the surgeon’s discretion.

In accordance with EAU guidelines for the adjuvant treatment of BCa, patients who are candidates for adjuvant chemotherapy/immunotherapy have pT3/t4 or N+ disease and are at high risk of BCa recurrence. Treatment is avoided if patients are at low risk of micrometastasis or recurrence. However, patients with other comorbidities such as renal insufficiency or heart disease are not candidates for adjuvant treatment. Specifically, in our study, of the 193 patients enrolled, 40 received adjuvant cisplatin treatment.

Variables collected include age, sex, smoking status, diabetes (blood glucose > 126 mg/dL), body mass index (BMI), clinical tumor (cT) stage, surgical approach, urinary diversion, pathological tumor (pT), nodal stage (pN), lymph vascular invasion (LVI), and adjuvant chemotherapy. Before RC, we conducted standard preoperative blood tests, clinical and physical examinations, and computer tomography (CT) scans to exclude the presence of metastases. A specialized uropathologist examined the RC specimen, staging them according to the Tumor Nodes Metastasis (TNM) classification system (2017 classification, 8th edition), and assessed tumor grade using the 2004/2016 World Health Organization system.

The SII value was obtained from the ratio of neutrophil × platelet/lymphocytes. The optimal cutoff value was defined by creating a receiver operating characteristics (ROC) curve with recurrence as the endpoint to yield the highest Youden index value. Our cohort of patients was divided into low SII and high SII groups according to SII value (<640.27 and >640.27, respectively).

### 2.2. Primary and Secondary Endpoints

The analysis’ primary endpoints were lymph node invasion (N), advanced pT stage (pT3/pT4), and locally advanced condition (defined as pT3/pT4 stage and/or nodal invasion) at RC pathology. Secondary endpoints of interest were recurrence-free survival (RFS), cancer-specific survival (CSS), and overall survival (OS). RFS was defined as the time between RC and the first local recurrence and/or distant metastasis. CSS and OS were defined as the time from RC to cancer-related death or the time from RC to any cause of death, respectively.

### 2.3. Statistical Analyses

The study included different steps. First, we divided our cohort according to the SII index (low SII group and high SII group). Descriptive analysis was presented using medians and interquartile ranges (IQR) for continuous variables, and frequencies along with percentages were provided for categorical variables. The Mann–Whitney *U* test was employed to compare continuous data, while categorical data were analyzed using either Chi-squared test (*X*^2^) or Fisher’s exact test. All tests were two-sided with a significant level set at *p* < 0.05. The study included different steps.

Second, univariable and multivariable logistic regression models using preoperative variables tested the association between SII and lymph node invasion (N), locally advanced disease (pT3/T4), and non-organ confined condition.

We evaluated the predictive performance of these models by calculating the area under the curve (AUC) of the ROC curve. We compared reference models that excluded SII with their AUCs using DeLong’s test to assess the additional prognostic value of pre-operative SII. We employed the Hosmer–Lemeshow goodness-of-fit test to examine the model’s performance.

Third, the Kaplan–Meyer estimates were used to evaluate RFS, CSS, and OS, and the log-rank method was used to determine significance.

Fourth, univariable and multivariable Cox proportional hazard regression models tested the relationship between the SII group with RFS, CSS, and OS. We assessed the discriminative performance of these models using Harrel’s concordance index (C-index). To evaluate the supplementary prognostic value of preoperative SII, we compared it with reference models that did not incorporate SII. Statistical significance was determined using the likelihood-ratio test. All statistical analyses were performed using STATA/SE version 18 (StataCorp, College Station, TX, USA).

## 3. Results

### 3.1. Main Features of Patients

**Figure 1:**
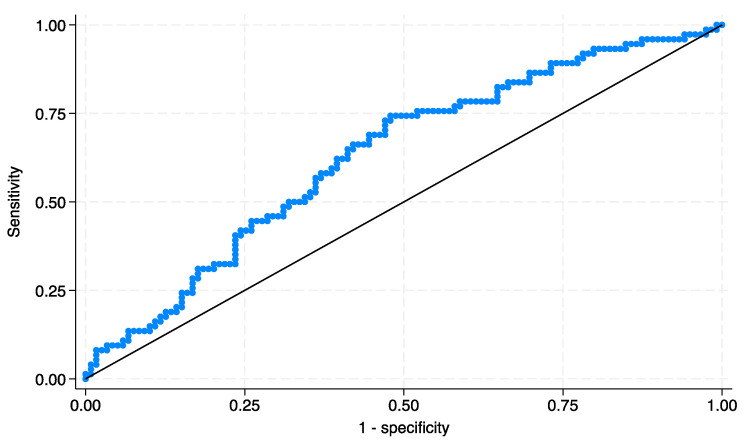
ROC curve for prediction of RFS in patients stratified by SII group.

The study encompassed a retrospective analysis of 193 patients. We found the best cutoff determined by the Youden index and the ROC analysis for RFS at 640.27 with an AUC equal to 0.64 (Figure 1). There were 80 (42%) patients with a low SII and 113 (58%) patients with a high SII. The demographic and descriptive features and laboratory data are reported in Table 1.

A high preoperative SII was more commonly observed in patients with disease in the severe clinical (*p* = 0.009) and advanced neoplastic stages (*p* = 0.005), nodal invasion (*p* = 0.002), lymph vascular invasion (*p* = 0.005), locally advanced disease (*p* < 0.001), progressive disease (*p* = 0.009), and all-cause deaths (*p* = 0.02).

### 3.2. Oncological Outcomes

Univariate and multivariate logistic regression analyses regarding the prediction of oncological outcomes are reported in Table 2. Increased SII was correlated with a higher probability of nodal invasion (odds ratio (OR) 3.76, 95% confidence interval (95% CI): 1.49–10.72; *p* = 0.002), advanced pT stage (pT3/pT4) (OR 2.26, 95% CI: 1.20–4.31; *p* = 0.006), and locally advanced condition (OR 2.92, 95% CI: 1.55–5.53; *p* < 0.001). With multivariate logistic regression models adjusted for the effects of standard preoperative characteristics (age, sex, smoking status, and clinical staging), a high SII remained independently linked to nodal invasion (OR 2.86, 95% CI: 1.07–7.59; *p* = 0.035), advanced pT stage (OR 2.17, 95% CI: 1.00–4.78; *p* = 0.048), and locally advanced condition (OR 2.81, 95% CI: 1.37–5.76; *p* = 0.005). The incorporation of SII in the reference model led to a statistically significant enhancement in its predictive validity for locally advanced condition (+5%, *p* = 0.04) but not for lymph node invasion (+3%, *p* = 0.19) or advanced pT stage (+2%, *p* = 0.13).

### 3.3. Survival Outcomes

The median follow-up duration was 20 months (IQR, 8–50). Patients in the high SII group had a notably shorter median follow-up period (16 vs. 37 months, *p* < 0.001). During the follow-up period, 74 (38.3%) patients had relapses, and 96 (49.7%) patients died, with 52 (26.9%) of these deaths attributed to cancer. High-SII patients, in comparison to their counterparts, had a higher incidence of recurrence (74.3% vs. 48.7%, *p* < 0.001), cancer-related deaths (65.3% vs. 56.0%, *p* = 0.24), and any-cause deaths (66.6% vs. 50.5%, *p* = 0.02).

**Figure 2:**
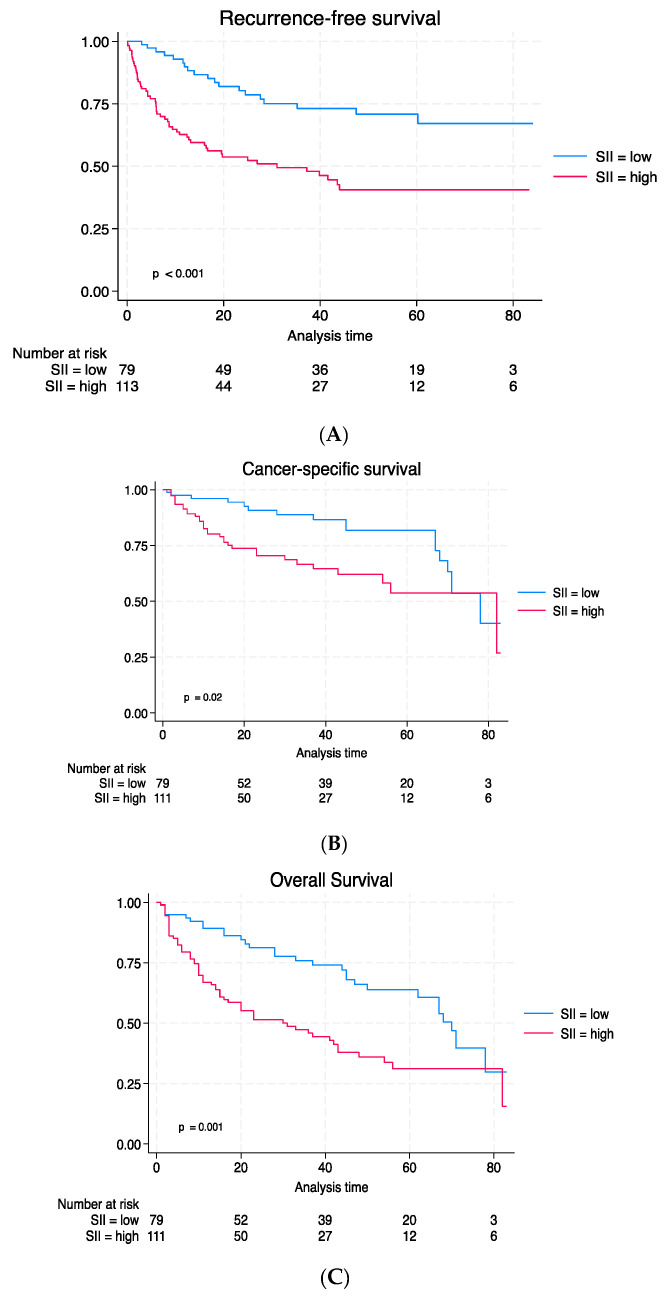
Kaplan–Meier estimates and log-rank of survival outcomes: (**A**) RFS; (**B**) CSS; and (**C**) OS.

The Kaplan–Meier curves illustrated notably worse survival results for recurrence-free survival (RFS) (Figure 2A), cancer-specific survival (CSS) (Figure 2B), and overall survival (OS) (Figure 2C).

In univariable Cox regression analyses, a high SII was linked to a higher risk of worse recurrence-free survival (RFS) (hazard ratio (HR) 2.82, 95% CI: 1.67–4.76; *p* < 0.001), cancer-specific survival (CSS) (HR: 1.86, 95% CI: 1.04–3.32; *p* = 0.035), and overall survival (OS) (HR: 2.01, 95% CI: 1.30–3.11; *p* = 0.002).

In multivariable Cox regression models, which accounted for clinicopathological preoperative factors, a high SII was significantly associated with RFS (HR: 2.15, 95% CI: 1.24–3.72; *p* = 0.007) and OS (HR: 1.72, 95% CI: 1.09–2.73; *p* = 0.018) (Table 3).

In multivariable Cox regression models, which were adjusted for postoperative features, an elevated SII was strongly associated with RFS (HR 2.1, 95% CI: 1.25–3.69; *p* = 0.004) and OS (HR: 1.80, 95% CI: 1.13–2.84; *p* = 0.012) but not with CSS. The inclusion of a preoperative SII in the reference model did not enhance the model’s discriminatory capacity for CSS (*p* = 0.08) but led to a statistically significant enhancement of RFS (+5%, *p* = 0.002) and OS (+5%, *p* = 0.01) (Table 4).

## 4. Discussion

This retrospective study demonstrated that a high elevated SII could be an independent predictor of aggressive BCa in patients treated with RC. We aimed to assess the potential of SII as a preoperative biomarker and further explore its association with histopathological outcomes through models incorporating preoperative and postoperative characteristics. According to the literature, a high SII was linked to aggressive disease (advanced clinical stage, lymphovascular invasion, and lymph node invasion) and remained independently associated with advanced pT stage, lymph node involvement, and locally advanced disease. In addition, we found that a high preoperative SII (>640.27) was independently correlated with survival outcomes (RFS and OS) in the model adjusted for both preoperative and postoperative features. To our knowledge, this study represents the first instance of such a correlation being observed within a logistic model incorporating postoperative characteristics. This result can potentially enhance predictive models, allowing for a more accurate prognosis in patients undergoing RC. Furthermore, these results showed the clinical importance of the immune system and the inflammatory response against cancer. Inflammation can influence different steps in carcinogenesis, starting with genetic mutations or alterations in gene regulation and then progressing to the onset, advancement, and metastasis of cancer [9,19].

As early as 1836, Rudolf Virchow demonstrated the link between inflammation and cancer, focusing on the role of the “reticular infiltrate” in certain types of chronic inflammation. Specifically, tumor-associated macrophages (TAM) and tumor-infiltrating T cells (TIL) are recruited by specific chemokines released by tumor cells. They can harm the vascular endothelium, break down the extracellular matrix, boost the proliferation of tumor cells, stimulate angiogenesis, and facilitate invasion and metastasis. In addition, the systemic release of cytokines and reactive oxygen species could lead to the modification of cytokine regulatory genes that are highly polymorphic and to mutagenic changes in DNA, which can damage certain repair proteins. So, if genetic damage is the “spark that ignites the fire of cancer “, certain inflammation might constitute the “fuel that feeds the flames”, contributing to making the tumor itself more aggressive [20].

In this way, the SII could be a real accessible marker used to assess the body’s overall inflammatory and immune responses, considering that this marker is affected by certain immune cells that play an important role in modulating the inflammatory response. Neutrophils can modulate the inflammatory response through the secretion of cytokines and chemokines. Tumors can influence the activity of neutrophils by releasing cytokines and chemokines [21,22,23]. When activated, neutrophils adhere to the endothelium, release proteolytic enzymes, and migrate through the basement membrane, representing the first line of defense against any external agent. When this activity diminishes (e.g., in neutrophilic conditions), the process of carcinogenesis prevails [24,25]. The second component of the SII index is the platelets. They release platelet-derived microvesicles (PMVs) that are able to interact with tumor cells and, more importantly, transfer miRNA inside the cancer cells, thereby promoting cancer progression [26]. The PMVs can also influence the neutrophils’ response, promote the diversification of macrophages in immune-active regions, and stimulate the emission of reactive oxygen species (ROS), thereby enhancing tumor activity [27,28]. The third cell type involved in the SII index is the lymphocytes. In many types of cancers, dysfunctional and exhausted CD8+ T cells have been found; this reduces the body’s defensive activity through the reduced formation of tumor necrosis factor (TNF), interleukin-2 (IL-2), and interferon-γ, which are important cytokines involved in the immune response [29,30].The interaction between the tumor microenvironment (TME) and CD8+ T lymphocytes favors the triggering of immunosuppressive mechanisms, including PD1 overexpression [31,32,33]. By evaluating the SII index, we can gain an overview of the mechanisms of the inflammatory response in the body. The higher the SII value, the greater the activity of procarcinogenic cells (neutrophils and platelets) and the lower the activity of defense cells (lymphocytes). The SII value has already been investigated by other groups, not only in urological cancer but also in other types of neoplasms. Wang et al. showed a connection between a high SII and lung cancer [34], and again Zhong et al. detected an association between a high SII and a worse prognosis in solid tumors [35]. A meta-analysis of 12 studies on the relationship between preoperative SII and urological cancers revealed that elevated SII levels were indicative of an unfavorable prognosis among these patients [36]. In bladder cancer in particular, Grossman et al. found that a high SII (>610) was correlated with poor OS, CSS, and any non-organ confined disease in a model based on preoperative characteristics but not on postoperative features [37]. In another study, Gorgel et al. reported that patients with an elevated SII (>843) exhibited significantly poorer CSS and OS in a model that considered clinicopathological features [38]. Unlike all the other studies, our study reveals an independent association between an elevated preoperative SII (>640.27) and both RFS and OS in models that incorporate both preoperative and postoperative features. In addition, in our logistic models, we have incorporated as many clinical details as possible that could be linked to the presence of more aggressive bladder cancer, such as smoking, which had not been previously investigated. This information could have the potential to enhance predictive models, allowing for a more accurate prognosis in patients undergoing RC. It should be pointed out, however, that despite these strong associations between SII and oncological outcomes, high SII values can be found in other pathological conditions, such as infections and autoimmune diseases, so we cannot yet consider this biomarker as a specific marker. It is essential to emphasize that simply observing a connection between a high SII and worse pathological and oncological outcomes in multivariable models does not automatically validate its clinical utility as a biomarker [39]. The primary importance of this study lies in its potential utility for clinicians as an additional marker for evaluating the prognosis of BCa. This can enhance risk assessment precision and aid in more accurate treatment planning decisions, including the consideration of adjuvant therapy. The use of these biomarkers may also be useful in identifying patients in need of new therapies, such as immune checkpoints inhibitors (ICIs). Guven et al. showed that a high baseline NLR and early NLR changes in patients treated with ICIs were associated with poor OS [40,41]. A PIV was also included in the prognostic scores of patients receiving treatment with ICIs, and again, higher values of these biomarkers were associated with lower OS and PFS and poorer responses to ICIs [42,43]. This research has limitations, including the retrospective cohort study was conducted at a single center and the relatively small patient sample size. The study’s strengths lie in the exclusion of potential confounding factors that might have been linked to SII, the same laboratory was used for the SII evaluations, and the same uropathologist performed histological examinations. Despite being similar to that used in other experiences, the best SII cutoff value remains unclear in BC patients. Further external validation with independent cohorts is needed to validate the generality of our findings. To confirm the general applicability of our findings, additional external validation using independent cohorts is required.

## 5. Conclusions

A high preoperative SII could be a real non-invasive, cheap, and easily obtainable predictive biomarker of aggressive disease in patients with bladder cancer. In our opinion, a single marker may not have sufficient predictive and prognostic power to be able to stratify patients according to their risk or enable personalized treatment strategies, but incorporating the SII into a regular monitoring protocol and including it in a panel of other markers or factors could increase our comprehensive understanding of individual patient risk. Sharing this knowledge about the SII index in daily clinical practice could be useful in identifying high-risk patients early, allowing for the use of targeted interventions and preventive measures (for instance, reducing the time from trans-urethral resection of the bladder to RC, intensifying radiological follow-up in high-risk patients, or proposing bladder sparing management in the lowest-risk patients).

## Figures and Tables

**Table 1:** Main patient features at RC stratified by SII group.

Characteristic	Total	Low SII	High SII	*p*
	N = 193	N = 80 (41.4)	N = 113 (58.5)	
Age, *n* (%)				0.82
<70	30 (15.5)	13 (12.4)	17 (17.6)	
≥70	163 (84.4)	67 (67.6)	96 (95.4)	
Sex, *n* (%)				0.06
Male	154 (79.7)	69 (86.2)	85 (75.2)	
Female	39 (20.2)	11 (13.7)	28 (24.7)	
Smoke, *n* (%)	149 (77.2)	65 (81.2)	84 (74.3)	0.25
Diabetes, *n* (%)	31 (16.0)	13 (16.2)	18 (15.9)	0.95
Clinical T stage, *n* (%)				**0.009**
cTa	67 (34.7)	32 (40.0)	35 (30.9)	
cTis	24 (12.4)	14 (17.5)	10 (8.8)	
cT1	49 (25.3)	19 (23.7)	30 (26.5)	
cT2	29 (15.0)	13 (16.2)	16 (14.1)	
cT3	18 (9.3)	2 (2.5)	16 (14.1)	
cT4	6 (3.1)	0 (0.0)	6 (5.3)	
BMI, median (IQR)	26 (24–29)	27 (25–29)	26 (24–28)	**0.04**
Surgical approach, *n* (%)				1.00
Open	186 (96.3)	77 (96.2)	109 (96.4)	
Robot-assisted	7 (3.6)	3 (3.7)	4 (3.5)	
Urinary diversion, *n* (%)				**0.005**
Ureterocutaneostomy	21 (10.8)	6 (7.5)	15 (13.2)	
Ileal conduit	148 (76.6)	57 (71.2)	91 (80.5)	
Orthotopic neobladder	24 (12.4)	17 (21.2)	7 (6.1)	
Pathological T stage, *n* (%)				**0.005**
pT0	14 (7.2)	6 (5.8)	8 (8.2)	
pTa	10 (5.1)	4 (4.1)	6 (5.9)	
pTis	23 (11.9)	14 (9.5)	9 (13.5)	
pT1	26 (13.4)	17 (10.8)	9 (15.2)	
pT2a	30 (15.5)	10 (12.4)	20 (17.6)	
pT2b	5 (2.5)	3 (2.1)	2 (2.9)	
pT3a	49 (25.3)	21 (20.3)	28 (28.7)	
pT3b	8 (4.1)	1 (3.3)	7 (4.7)	
pT4a	21 (10.8)	4 (8.7)	17 (12.3)	
pT4b	7 (3.6)	0 (2.9)	7 (4.1)	
N, *n* (%)	37 (19.1)	7 (8.7)	30 (26.5)	**0.002**
LVI, *n* (%)	128 (66.3)	44 (55.0)	84 (74.3)	**0.005**
Locally advanced disease, *n* (%)	102 (52.8)	30 (37.5)	72 (63.7)	**<0.001**
Adjuvant chemotherapy, *n* (%)	40 (20.7)	14 (17.5)	26 (23.0)	0.35
Progressive disease, *n* (%)	61 (31.6)	17 (21.2)	44 (38.9)	**0.009**
Any-cause deaths, *n* (%)	96 (49.7)	32 (40.0)	64 (56.6)	**0.02**
Cancer-related deaths, *n* (%)	52 (26.9)	18 (22.5)	34 (30.0)	0.24

SII = Systemic immune-inflammation index; BMI = Body mass index; IQR = Interquartile range; LVI = Lymph vascular involvement; N = Lymph node invasion. Bold: emphasise the statistical correlation between the calculations.

**Table 2:** Multivariate logistic regression analyses predicting oncological outcomes.

Characteristic	OR	N			Advanced pT Stage			Locally Advanced Condition	
		95% CI	*p*-Value	OR	95% CI	*p*-Value	OR	95%CI	*p*-Value
SII (Reference:low)									
High	2.86	1.07, 7.59	**0.035**	2.17	1.00, 4.78	**0.048**	2.81	1.37, 5.76	**0.005**
Age	1.10	0.94, 1.04	0.89	0.83	0.31, 2.26	0.728	1.05	0.41, 2.67	0.90
Smoke (Reference: no)									
Smoke	0.56	0.22, 1.41	0.21	0.67	0.26, 1.67	0.392	1.28	0.52, 3.14	0.57
Sex (Reference: male)									
Female	0.53	0.18, 1.52	0.23	0.25	0.08, 0.80	**0.019**	0.52	0.19, 1.38	0.19
Clinical tumor stage									
(Reference: cTa/cTis/cT1)									
cT2	5.38	1.97, 14.62	**0.001**	56.10	11.42, 275.61	**<0.001**	31.06	6.68, 144.34	**<0.001**
cT3/cT4	10.69	3.75, 30.47	**<0.001**	33.42	6.78, 164.58	**<0.001**			
Goodness-of-fit test	Hosmer–Lemeshow test		0.89			0.18			0.85
AUC									
Model with SII		AUC: 0.79			AUC: 0.81	0.26		AUC: 0.81	
Model without SII		AUC: 0.76 (+3%)			AUC: 0.79 (+2%)			AUC: 0.76 (+5%)	
(*p* = difference model)			0.19			0.13			**0.04**

SII = Systemic immune-inflammation index; N = Lymph node invasion; OR = Odds ratio; CI = Confidence interval; AUC = Area under the curve. Bold: emphasise the statistical correlation between the calculations.

**Table 3:** Preoperative multivariate Cox regression analyses for prediction of RFS, CSS, and OS.

	Recurrence-Free Survival			Cancer-Specific Survival			Overall Survival		
	HR	95% CI	*p*-Value	HR	95%CI	*p*-Value	HR	95%CI	*p*-Value
SII (Reference: low)									
High	2.11	1.22, 3.66	**0.007**	1.33	0.71, 2.51	0.369	1.73	1.09, 2.74	**0.018**
Age	0.79	0.41, 1.50	0.481	1.30	0.54, 3.11	0.550	1.16	0.64, 2.12	0.607
Smoke (Reference: no)									
Smoke	0.88	0.52, 1.48	0.633	1.32	0.64, 2.73	0.450	1.19	0.71, 1.99	0.500
Gender (Reference: male)									
Female	0.85	0.48, 1.50	0.594	1.45	0.76, 2.74	0.253	1.06	0.64, 1.75	0.809
Clinical tumor stage									
(Reference: cTa/cTis/cT1)									
cT2	1.63	0.85, 3.15	0.140	1.09	0.44, 2.68	0.849	1.32	0.73, 2.39	0.356
cT3/cT4	7.33	4.05, 13.27	**<0.001**	8.18	3.91, 17.08	**<0.001**	4.05	2.28, 7.21	**<0.001**
C-index									
Model with SII			0.73			0.74			0.68
Model without SII			0.68 (+5%)			0.72 (+2%)			0.63 (+5%)
(*p* = difference model)			**0.005**			0.36			**0.016**

HR = Hazard ratio; CI = Confidence interval; SII = Systemic immune-inflammation index; Bold: emphasise the statistical correlation between the calculations.

**Table 4:** Postoperative multivariate Cox regression analyses for prediction of RFS, CSS, and OS.

Characteristic	Recurrence-Free Survival			Cancer-Specific Survival			Overall Survival		
	HR	95%CI	*p*-Value	HR	95%CI	*p*-Value	HR	95%CI	*p*-Value
SII (Reference: low)									
High	2.20	1.28, 3.78	**0.004**	1.65	0.89, 3.08	0.111	1.79	1.13, 2.84	**0.012**
Age	0.97	0.51, 1.83	0.926	1.52	0.64, 3.64	0.338	1.25	0.68, 2.72	0.462
Smoke (Reference: no)									
Smoke	1.22	0.71, 2.12	0.462	2.06	0.99, 4.29	0.052	1.68	0.99, 2.26	**0.050**
Gender (Reference: male)									
Female	1.47	0.80, 2.70	0.213	1.80	0.93, 3.48	0.076	1.35	0.80, 2.26	0.252
Pathological tumor stage									
(Reference: pT0/pTa/pTispT1)									
pT2	1.58	0.62, 4.01	0.331	1.00	0.32, 3.15	0.992	1.50	0.68, 3.29	0.304
pT3/pT4	2.92	1.18, 7.23	**0.020**	3.03	1.21, 7.59	**0.017**	3.33	1.63, 6.78	**0.001**
Lymphovascular invasion	1.02	0.41, 2.48	**0.05**	0.94	1.75, 8.86	0.903	0.92	0.46, 1.82	0.828
Lymph node invasion	3.63	2.02, 6.50	**<0.001**	3.94	1.75, 8.86	**0.001**	2.91	1.63, 5.19	**<0.001**
Adjuvant chemotherapy	1.50	0.85, 2.63	0.157	0.48	0.21, 1.10	0.086	0.67	0.38, 1.19	0.176
C-index									
Model with SII			0.79			0.76			0.74
Model without SII			0.77 (+2%)			0.76			0.73 (1%)
(*p* = difference model)			**0.002**			0.08			**0.01**

HR = Hazard ratio; CI = Confidence interval; SII = Systemic immune-inflammation index; Bold: the statistical correlation between the calculations.

## Data Availability

All data generated for this analysis were from an anonymized database. The code for the analyses will be made available upon request.
